# Intramedullary Bone Lengthening Following Preceding Hip Surgery—A Case Series

**DOI:** 10.3390/jcm9124104

**Published:** 2020-12-19

**Authors:** Lukas Zak, Thomas Manfred Tiefenboeck, Gerald Eliot Wozasek

**Affiliations:** Department of Orthopedics and Trauma-Surgery, Medical University of Vienna, Trauma Surgery, Waehringer Guertel 18–20, A-1090 Vienna, Austria; thomas.tiefenboeck@meduniwien.ac.at (T.M.T.); gerald.wozasek@meduniwien.ac.at (G.E.W.)

**Keywords:** limb lengthening, intramedullary lengthening, hip surgery, limb length discrepancy

## Abstract

Limb length discrepancy (LLD) is a common problem after joint-preserving hip surgeries, hip dysplasia, and hip deformities. Limping, pain, sciatica, paresthesia, and hip instability are common clinical findings and may necessitate limb-lengthening procedures. The study included five patients (two female and three male, mean age of 28 years (20–49; SD: 12)) with symptomatic limb length discrepancy greater than 2.5 cm (mean: 3.6 cm) after total hip arthroplasty (THA), hip dysplasia, or post-traumatic hip surgery. They underwent either ipsi- or contralateral intramedullary limb-lengthening surgeries using the PRECICE™ telescopic nail. All patients achieved complete bone healing and correction of the pelvic obliquity after intramedullary lengthening. None of the patients had a loss of proximal or distal joint motion. The mean distraction-consolidation time (DCT) was 3.8 months, the distraction index (DI) 0.7 mm/day, the lengthening index (LI) 1.8 months/cm, the consolidation index (CI) 49.2 days/cm, the healing index (HI) 1.1 months/cm, and the modified healing index (HI*) 34 days/cm. Intramedullary limb lengthening after LLD in cases of hip dysplasia, hip deformity, and various kinds of hip surgery is a useful and safe procedure in young patients to achieve equal limb length. No functional impairment of the preceded hip surgery was seen.

## 1. Introduction

Limb length discrepancy (LLD) is a common problem after hip surgeries, hip dysplasia, and hip deformities [1,2,3,4,5,6,7,8]. It is often associated with low back pain, sciatica, hip pain syndromes, hip instability, limited walking capacity, limping, and reduced patient satisfaction [5,9,10]. After total hip arthroplasty, an LLD between 3 and 70 mm was reported in 1% to 27% of all cases [1,7]. This issue can arise from cup malposition, inequalities in bony structure, pelvic tilt, and a more inferior change in the original acetabulum [8]. If clinical symptoms occur, revision surgeries such as acetabular revisions, modular femoral head exchanges, and femoral component revisions can be necessary [5].

LLD after per- and intertrochanteric fracture treatment or joint-preserving hip surgery for femoral neck fractures is a common clinical finding in nongeriatric patients [2,6]. Depending on the fracture type and treatment, most patients presented LLD < 2 cm after per- and intertrochanteric fractures, but 7.4% of all surgeries resulted in differences of 2 cm and above [6]. After femoral neck fractures, joint-preserving hip surgery shortened the limb by more than 1 cm in one-third of patients, but a difference greater than 2 cm was not observed [2]. In contrast, dysplasia of the hip and its complications can result in an LLD of up to 5 cm [3,4].

Bony, functional, and anatomical LLD has to be differentiated and can be classified into three categories: shortening less than 2 cm, which is usually not clinically relevant and can be ignored; 2–4 cm, with the possibility of lengthening; more than 4 cm, where lengthening is needed to avoid possible complications of lower limb length inequality such as pelvic obliquity and scoliosis [11]. 

Intramedullary limb lengthening is an attractive and effective treatment method with significant advantages to external fixation. Patients report better overall satisfaction, reduced discomfort, and more effective rehabilitation as fewer limitations in physiotherapy appear [12,13,14,15]. 

This case series presents LLD treatment options in primary hip diseases after preceding hip surgery. We hypothesized that intramedullary lengthening is an appropriate tool. 

## 2. Patients and Methods

This retrospective case series includes patients who had intramedullary limb-lengthening surgeries on the femur to correct a symptomatic limb-length discrepancy following primary hip disease, fractures around the hip joint, hip surgery, or pelvic correction surgery. Surgeries were performed by two surgeons between 2016 and 2019 in a single academic trauma-surgery center. 

Excluded were all patients who underwent intramedullary lengthening on the lower limb with intact, nonpathological hip joints and cases with pathologic hips but concomitant injuries on the lower limb. 

All procedures involving human participants were in accordance with the ethical standards of the institutional ethical review board (Nr. 1573/2020) and with the 1964 Helsinki Declaration and its later amendments or comparable ethical standards. 

### 2.1. Surgery

All patients were assessed preoperatively, including a thorough physical examination, especially of the hip joints. Movement and stability were evaluated to exclude the necessity of local hip revision surgery. Current radiographs, including long-standing radiographs with reference markers, were performed. LLD was measured with our radiologic picture archiving and communication program (IMPAX EE, Agfa HealthCare Corporation, Bonn, Germany), and TraumaCad software (Brainlab Ltd., Petach-Tikva, Israel) was used for preoperative planning. 

The decision between antegrade and retrograde lengthening was made depending on the trochanteric deformity. While antegrade nailing was preferred to prevent damage at the knee joint, a retrograde approach was used in cases with ipsilateral dysplasia. 

After osteotomy with a drill and a chisel, a magnetically actuated intramedullary nail (P2, PRECICE, NuVasive Inc., San Diego, CA, USA) was inserted for bone lengthening. The antegrade femoral nails had a 10° proximal bend and were used at a trochanteric entry point for insertion. The 10° bent retrograde femoral nails were implanted at the routine entry point. A pin for rotational guidance and a screw for reamer guidance were used if necessary. We performed osteotomy typically with drill and chisel.

### 2.2. Postoperative Care

Patients were taught how to use the remote control to lengthen the nail. Physiotherapy (gait training and physical exercises) was initiated during the hospital stay. Weight-bearing of up to 5 kg was permitted immediately after surgery. Actual lengthening was started five to seven days after surgery with a distraction rate of 0.66 mm/day (two times 0.33 mm/day) up to 1 mm/day (three times 0.33 mm/day), depending on the occurrence of pain and adjacent joint range of motion. Patients were followed up every second week during lengthening and every fourth to sixth week during the consolidation phase. Regular physiotherapy, daily training on the continuous passive motion device, and daily home exercises with the main emphasis on active and passive extension of the knee were recommended. We permitted full weight-bearing as soon as radiographs showed callus formation in the distraction gap on three out of four cortices. During lengthening and consolidation, vitamin D3 and calcium were substituted orally. 

Complications were classified according to Paley et al., taking the modification for intramedullary lengthening of Schiedel et al. into account [14,16].

A problem was defined as a potential expected difficulty during implantation, the latency phase, and the distraction or fixation period. It should be fully resolved by the end of the treatment period without surgery. In contrast, for an obstacle, a surgical intervention under anesthesia becomes necessary. Any local or systemic intra- or perioperative complications, difficulties during distraction or consolidation that remained unsolved at the end of the treatment period, as well as early or late post-treatment difficulties, were considered as true complications. 

### 2.3. Distraction Parameters

We determined the distraction-consolidation time (DCT), defined as the time interval in months from the start of distraction to callus formation. Complete consolidation was defined by the presence of three out of four continuous cortices in two X-ray projections. The healing index (HI) was described as the ratio of DCT per cm lengthening (months/cm). However, we also assessed a modified healing index (HI*) by DeBastiani and Aldegheri, representing the number of days in an external distraction frame per centimeter gain in length, as a valid and reliable quantitative indicator of the potential for bone formation [17,18]. We adapted both indices to the technique of intramedullary lengthening. 

A further used parameter was the distraction index (DI), described as the distance of length achieved in millimeters divided by the duration of lengthening in days (mm/days). The consolidation index (CI) was defined as the number of days from surgery until consolidation divided by the length of the regenerate in centimeter (days/cm). The lengthening index (LI) refers to the number of months an external fixator is mounted per one-centimeter lengthening [19]. For intramedullary lengthening, LI was adapted to represent the number of months until full weight-bearing was allowed, and complete bone healing was achieved.

### 2.4. Clinical Evaluation

According to the modified classification of the Association for the Study and Application of the Method of Ilizarov (ASAMI), the bone results were based on four criteria, including union, infection, deformity, and leg length discrepancy. Functional outcomes were based on the following five criteria: the presence of a limp, stiffness of the adjacent joints, pain, soft tissue sympathetic dysfunction, and the ability to perform previous activities of daily living (ADL) [20]. Hip, knee, and ankle function were assessed pre- and postoperatively. 

### 2.5. Statistical Analysis

Descriptive data (mean, range, standard deviation) were reported for the entire patient cohort. Statistical analysis focused on surgical, radiological, and functional outcomes. Therapeutic variables (surgery, function, and distraction parameters), pathological variables (complications), and demographic variables (sex, age, etc.) were examined. All calculations were made using Microsoft Excel^®^.

## 3. Results

### 3.1. Case Histories

**Figure 1:**
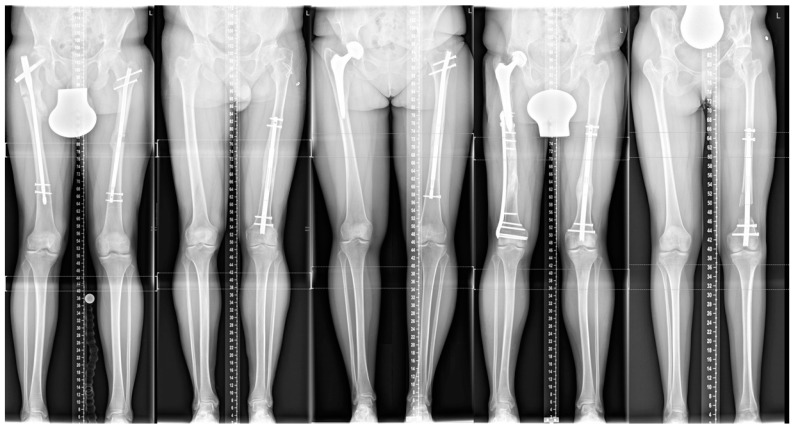
Long-standing radiographs are presenting all five patients (1 to 5).

After applying the exclusion criteria, five patients (two men and three women) with a mean age of 28 years and a mean LLD of 36 mm were left for final analysis. Demographic data and baseline characteristics are presented in Table 1, a patient’s list in Table 2. Figure 1 shows the patient’s X-rays.

Case 1:

The male patient suffered from femoral and tibial cyst treatment with homologous bone filling abroad in childhood, followed by a pericystic fracture and re-filling of the cyst at 14. The patient sustained a subtrochanteric, pericystic fracture at the age of 19, stabilized with a long nail. After fracture healing, he complained of a 2.5 cm LLD with contralateral shortening, leading to abnormal walk and low back pain. Treatment involved an antegrade femoral lengthening nail in achieving equal limb length.

Case 2:

The 49-year-old male initially presented with left hip dysplasia and symptomatic LLD. He had previously undergone left femoral correction surgery at the age of 30. He was limping and developed symptoms such as sciatic pain and back pain. A 5 cm shortening on the ipsilateral side was corrected by retrograde femoral lengthening. The patient was satisfied with the result with no restrictions in activities of daily living.

Case 3:

The now 28-year-old female patient was treated with total hip arthroplasty (THA) after Perthes disease at 23. Shoe-lifts could partially correct a contralateral shortening of 2.5 cm. She complained about low back pain and symptomatic scoliosis. After detailed examination and surgical planning, an antegrade femoral nail was implanted for 2.5 cm lengthening.

Case 4:

The male patient with hip dysplasia on both sides underwent THA at the age of 15, with a resulting LLD of 1 cm after multiple surgeries. The patient was injured in a car accident at 17 and suffered from a comminuted periprosthetic open fracture, which was treated with open reduction and internal plate fixation. The bone healed with an LLD of 2.5 cm. He started to complain of limping and needed to wear shoe-lifts at his young age. Therefore, lengthening surgery was performed at the age of 20. The hip dysplasia on the shorter leg was untreated and asymptomatic. We achieved equal limb length and uneventful bone healing with high patient satisfaction.

Case 5:

The 23-year-old patient was surgically treated with a congenital heart defect in early childhood. She suffered from bilateral hip dysplasia, primarily treated with a pelvic correction surgery on the right side, resulting in a 5 cm shortening. The patient’s height was 149 cm, and she complained about low back pain caused by scoliosis and severe limping. A retrograde telescopic nail was inserted after distal femoral osteotomy for intramedullary lengthening. One year after surgery, bone healing was still insufficient. The nail broke at the distal interlocking bolt. Revision surgery was done with a solid nail, combined with an autologous bone graft from the iliac crest for further uneventful bone healing. 

### 3.2. Demographic Results 

Symptomatic LLD was affected by trauma in two cases, including a pericystic fracture and a periprosthetic fracture. Reasons for LLD were ipsilateral shortening after hip correction surgery or pelvic correction surgery and lengthening after total hip replacement or fracture treatment. Two antegrade and three retrograde nails were implanted in the shorter limp—three on the more-or-less unaffected bone, two on the affected bone. 

The five patients underwent seven previous surgeries around the hip joint—at least one for each patient. No comorbidities were described. Two out of five patients declared to smoke 10 to 20 cigarettes per day. After a mean maximal follow-up time of 27 months, two out of five nails were removed. 

### 3.3. Distraction Parameters and Functional Results

The mean DCT was 3.8 months, the DI 0.7 mm/day, the LI 1.8 month/cm, the CI 49.2 days/cm, the HI 1.1 months/cm, and the HI* 34 days/cm. The results of the distraction indices are presented in Table 3. 

ASAMI bone and ASAMI function were graded excellent in all cases post-op after complete healing. No patient had a loss of proximal or distal joint motion at the time point of complete bone healing. 

### 3.4. Complications and Problems

According to Paley’s classification, only one true complication was reported. Complete bone healing was missing in patient five during the consolidation phase. Full weight-bearing caused a nail breakage of the 40 kg female, nonsmoking patient, even before revision surgery was planned. The nail was removed and exchanged for a solid trauma nail, combined with an autologous bone graft of the contralateral iliac crest. 

One problem occurred, as a clinical overlengthening of 5 mm was corrected by backwinding the nail with the remote control without any difficulties in further bone healing. This is an advantage of the used nail. 

## 4. Discussion

In our study, five femoral intramedullary lengthening cases are presented with various previous correction surgeries and a remaining LLD of more than 2.5 cm. In all patients, leg length equality and complete bone healing were achieved after a mean lengthening time of 202 days. All patients reported high satisfaction and were able to return to daily life activities. They were able to walk without shoe lifts or any other devices. LLD is a common problem in primary hip diseases and after surgeries around the hip. In most cases, conservative management will lead to satisfactory clinical results. However, when symptoms appear, surgical treatment should be considered.

This study is one of the first investigating ipsi- or contralateral lengthening after prior correction surgery to the structures around the hip. 

Physical examination is of outstanding importance to rule out other causes of deformity and LLD [21,22]. In minor LLD cases with minimal symptoms, patients may benefit from conservative treatment. However, an LLD >20 mm may indicate surgery [10].

Various approaches are described to correct LLD after surgeries around the hip. Length correction by THA is, however, limited to 4 cm, as acute correction can cause nerve palsy [21]. Two-stage THA with primary extensive soft tissue release and skeletal traction, followed by THA, can be an option in highly selected patients [23]. An alternative is contralateral limb shortening [24], which is indicated when lengthening is not possible—for example, after long-stem THA [25] or long-stem total knee arthroplasty. Epiphysiodesis is the method of choice in the juvenile population [3]. 

To our knowledge, only a few studies have addressed this topic. Harkin et al. published three hip dysplasia cases treated by THA and retrograde intramedullary femoral lengthening in a staged procedure [21]. They reported excellent outcomes regarding bone healing, leg length equality, and patient comfort [21]. We stated the same satisfactory results, except a nail breakage after missing bone healing one year after surgery. 

Thakral et al. found (contralateral) limb lengthening for LLD after THA to be a safe and effective treatment when conservative treatment was unsuccessful. They presented the technique of lengthening over a femoral nail with an external fixator in one case and lengthening with an intramedullary kinetic skeletal distractor in two cases. All treatments were successful regarding lengthening, bone healing, and progressing to weight-bearing [26]. The mean lengthening distance of 2.7 cm was shorter than the mean of 3.6 cm in our series, which is typical for symptomatic THA patients. Their results are comparable to ours.

Yoshida et al. reported two cases of osteoarthritis due to functional acetabular dysplasia caused by LLD. They used a hexapod ring fixator to correct insufficient acetabular coverage of the femoral head and lateral inclination of the pelvis [27]. This overuse and pathological stress on the hip joints must be avoided, especially in cases with “pre-damaged” hip joints. Equal limb length should be aimed in all cases of joint-preserving surgeries. 

This study’s limitation is the small patient size of this nonconsecutive case series, which is typical on this specific topic. A comparison group is missing. The presented patients were treated in a trauma unit with high external and internal bone lengthening and deformity correction experience but less experience in hip dysplasia surgery.

## 5. Conclusions 

Intramedullary femoral lengthening after operatively treated hip dysplasia, hip deformity, and hip surgery appeared to be a safe and effective treatment method for large LLD in carefully selected cases. After reaching leg length equality, all patients could return to activities of daily living, and all patients reported improved satisfaction. 

## Figures and Tables

**Table 1:** Demographic data and baseline characteristics.

Variables	
Demographic data	Total
Patients	5
Age (years)	28 (20–49, SD 12)
Sex (F/M)	2/3
Baseline characteristics	
Side (right/left)	1/4
Localization	
Femur antegrade	2
Femur retrograde	3
Lengthening (mm)	36 mm (25–50, SD 13)

**Table 2:** Patient list presenting patient characteristics, primary disease, previous surgery, and treatment. Ante: antegrade insertion; retro: retrograde insertion; fx: fracture, re-fx: refracture; THR: total hip replacement.

Patient List									
ID	gender	age	side	Nail	insert	primary disease	surgery	Trauma + treatment	shortening
1	male	20	right	P2	ante	trochanteric bone cyst	bone filling after fx	pertrochanteric re-fx + fixation	contralateral
2	male	49	left	P2	retro	hip dysplasia	Femoral correction s.		ipsilateral
3	female	28	left	P2	ante	M. Perthes	THR		contralateral
4	male	22	left	P2	retro	hip dysplasia on both sides	THR right right	periprosthetic fracture + plate	contralateral
5	female	23	left	P2	retro	hip dysplasia on both sides	pelvic correction surgery		ipsilateral

**Table 3:** Patient list with distraction parameters. LD: lengthening distance in cm; LI: lengthening index; DI: distraction index; CI: consolidation index; DCT: distraction-consolidation time; HI: healing index; HI*: modified healing index.

Distraction Parameters									
ID	Gender	Age	LD	LI	DI	CI	DCT	HI	HI*
1	male	20	2.5	2.3	0.6	71.5	3.7	1.8	56.0
2	male	49	5	1.7	0.7	50.6	6.0	1.2	36.4
3	female	28	2.5	1.2	0.6	35.6	1.5	0.6	18.4
4	male	22	3	1.3	0.9	39.3	2.8	0.9	28.7
5	female	23	5	2.7	0.6	82	11.0	2.2	66
